# Interference and interferometry in electron holography

**DOI:** 10.1093/jmicro/dfaa033

**Published:** 2020-06-26

**Authors:** Ken Harada

**Affiliations:** CEMS, RIKEN (The Institute of Physical and Chemical Research), Hatoyama, Saitama 350-0395, Japan

**Keywords:** electron wave, electron holography, transmission electron microscopy, interference microscopy, phase, wavefront, reconstruction

## Abstract

This paper reviews the basics of electron holography as an introduction of the holography part of this special issue in Microscopy. We discuss the general principle of holography and interferometry regarding measurements and analyses of phase distributions, first using the optical holography. Next, we discuss physical phenomena peculiar to electron waves that cannot be realized by light waves and principles of electromagnetic field detection and observation methods. Furthermore, we discuss the interference optical systems of the electron waves and their features, and methods of reconstruction of the phase information from electron holograms, which are essential for realization of practical electron holography. We note that following this review application of electron holography will be discussed in detail in the papers of this special issue.

## Introduction

Techniques for measuring and visualizing the phase distribution of waves are called the phase problem. This is a new as well as old problem not only for electron waves but also for light waves and X-rays, and various methods have been developed and examined. Among these methods, electron holography [1, 2] is the only method that can deterministically measure the phase distributions based on the phase of the reference wave.

Electron holography was invented in 1947 by Gabor [3, 4] as one of the aberration correction methods for objective lenses of electron microscopes. At that time, there were several trials to advance electron interferometry [5–8]; however, it was difficult to obtain electron beams having sufficient coherence. In 1962 holography was first put to practical use by employing a laser beam by Leith and Upatnieks [9]. In the optical microscopy, however, lens aberrations can be corrected by using the lens itself, and consequently holography was mostly used as three-dimensional imaging [10] and other applications, such as measurement of Poisson’s ratio [11].

In 1978 Tonomura et al. realized a practical-level electron holography using a field-emission transmission electron microscopy (TEM) with an acceleration voltage of 70 kV [12]. After that, electron holography was used not only to show follow-up experiments [13, 14] confirming interference phenomena in light optics but also to observe electromagnetic properties of materials [15] that cannot be achieved using light. Specific examples include confirmation experiments of the Aharonov-Bohm effect [16, 17] and observations of superconducting magnetic flux quanta [18–22].

Recently, thanks to technology advancement, a lot of techniques and instruments for electron holography and interferometry have been developed: electron beams (or waves) with sufficient coherence to carry out electron holography are provided by field-emission electron sources [23, 24], beam splitters that are essential for interferometry by superimposing waves are realized by use of electron biprisms [25], high-resolution fringe detection and recording in the electron interferometry are accomplished by charge-coupled device (CCD) cameras and direct electron detection (DED) cameras [26], and commercial-level reconstruction and analyses of recorded holograms can be numerically performed by using developed algorithms and softwares.

This paper reviews electron holography and its optics and interferometers together with its physical backgrounds. It focuses on the basic parts so that it can easily be understood by researchers who are not familiar with electron holography. Recent results of various applications are discussed by experts in the papers to follow in this issue. These include electromagnetic fields, magnetizations, inner-potentials and dielectric polarizations in materials, atomic-level high-resolution observations and strain field observations.

## Principle of holography

Understanding holography requires knowledge in wave optics such as propagation, diffraction and interference. Holography can be realized not only by visible light waves but also by microwaves, supersonic waves and electron waves. The principle of holography of light waves is often applied to the principle of other wave holography. In this paper, we discuss electron holography; first, two-wave interference in terms of off-axis holography is described following the discussion of light-wave holography [27, 28]. Then, we discuss wavefront-division-type holography as a practical example of electron holography techniques.

### Two-wave interferometry as holography

**Fig. 1:**
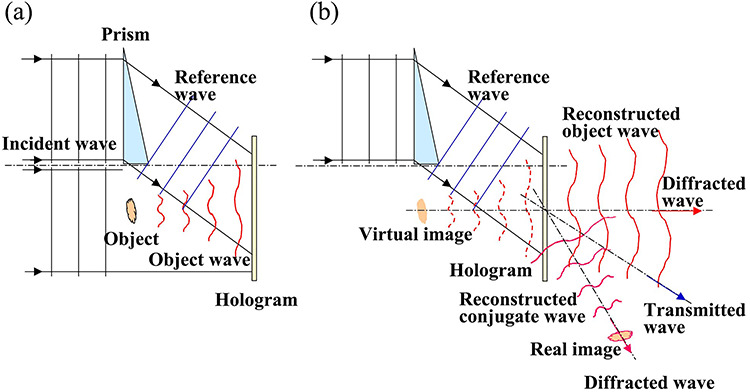
Principle of off-axis holography: (a) recording of hologram and (b) reconstruction from hologram.

Holography requires two-step procedures: (i) recording holograms and (ii) reconstructing image data to retrieve phase information of the waves from the hologram [27, 28]. Figure 1 shows a schematic diagram of the off-axis and Fresnel holography without any optical devices placed between the object and the hologram. Figure 1a shows how to record object waves as holograms, and Fig. 1b shows how to reconstruct object waves from the holograms.

In Fig. 1a, an object wave transmitted through an object and/or scattered at the object and a reference wave propagating without any scattering are superimposed and interfered. The film on which the interference pattern is recorded or the interference pattern itself is called a hologram. To reconstruct the object wave from the hologram, another wave is irradiated on the hologram, and one of the diffracted waves is chosen and imaged as the reconstructed object wave.

Figure 1b shows two waves diffracted from the hologram and a transmitted wave. One wave represents a reconstructed object wave forming a virtual image of the object, and another wave represents a conjugate wave forming a real image. This is an advantage of the off-axis holography in that these two images are reconstructed at different places with different propagating directions, solving the disadvantage of the in-line holography named ‘twin image’, so that we can use either one of the images for analysis [9, 10].

To describe this electron wave behavior, we introduce the following mathematical expressions. By defining the wavelength by *λ*, the object wave by Φ_Obj_ (*x*, *y*), the reference wave by Φ_Ref_ (*x*, *y*), the respective amplitude distributions by *φ*_Obj_ (*x*, *y*) and *φ*_Ref_ (*x*, *y*) and the respective phase distributions by *η*_Obj_ (*x*, *y*) and *η*_Ref_ (*x*, *y*), we can write down the object wave Φ_Obj_ (*x*, *y*) and the reference wave Φ_Ref_ (*x*, *y*) as

(1) \begin{equation*} {\Phi}_{\mathrm{Obj}}\big(x,y\big)={\varphi}_{\mathrm{Obj}}\big(x,y\big)\mathit{\exp}\big[i{\eta}_{\mathrm{Obj}}(x,y)\big], \end{equation*}

(2) \begin{equation*} {\Phi}_{\mathrm{Ref}}\big(x,y\big)={\varphi}_{\mathrm{Ref}}\big(x,y\big)\mathit{\exp}\big[i{\eta}_{\mathrm{Ref}}\big(x,y\big)\big]. \end{equation*}

Using these we obtain the intensity distribution (hologram) *I*_Holo_ (*x*, *y*) generated by interference of the two waves as

$$\kern-6pc {I}_{\mathrm{Holo}}\big(x,y\big)={\big|{\varphi}_{\mathrm{Obj}}\big(x,y\big)\big|}^2+{\big|{\varphi}_{\mathrm{Ref}}\big(x,y\big)\big|}^2 $$

(3) \begin{equation*} +{\varphi}_{\mathrm{Obj}}^{\ast}\big(x,y\big){\varphi}_{\mathrm{Ref}}\big(x,y\big)\exp \big[-i\big({\eta}_{\mathrm{Obj}}\big(x,y\big)-{\eta}_{\mathrm{Ref}}\big(x,y\big)\big)\big] \end{equation*}

$$ \kern-1.5pc+ {\varphi}_{\mathrm{Obj}}\big(x,y\big){\varphi}_{\mathrm{Ref}}^{\ast}\big(x,y\big)\exp \big[i\big({\eta}_{\mathrm{Obj}}\big(x,y\big)-{\eta}_{\mathrm{Ref}}\big(x,y\big)\big)\big]. $$

For simplicity, in the following we assume that the object wave propagates in parallel with the optical axis and the reference wave is tilted by an angle *α* in the *x*-direction. In addition, we assume that the amplitude of the object wave with *φ*_Obj_ (*x*, *y*) is real and the reference wave is a plane wave with *φ*_Ref_ (*x*, *y*) = 1. Then, the intensity, i.e. an electron hologram, is expressed by

(4) \begin{eqnarray*} {I}_{\mathrm{Holo}}\big(x,y\big)\!\!\!&=&\!\!\!{\big|{\varphi}_{\mathrm{Obj}}\big(x,y\big)\big|}^2+1 \nonumber \\ &&\!\!\!+2{\varphi}_{\mathrm{Obj}}\big(x,y\big)\cos \big[{\eta}_{\mathrm{Obj}}\big(x,y\big)-2\pi{R}_{0x}x\big], \end{eqnarray*}

where *R*_0*x*_ (= (sin*α*)/*λ*) is the carrier spatial frequency.

In electron holography, an objective lens is placed between the object and hologram, and therefore the intensity distribution |*φ*_Obj_ (*x*, *y*)|^2^ represents the object image. As shown in Eq. (4), the hologram *I*_Holo_ (*x*, *y*) consists of |*φ*_Obj_ (*x*, *y*)|^2^ and the two-wave interference fringes created by the object wave and the reference wave with the averaged spacing 1/*R*_0*x*_. This object image superimposed with the interference fringes, i.e. an interferogram or an image hologram, characterizes the electron holography.

The hologram represented by Eq. (4) is reconstructed by several methods which will be discussed later. The component by the tilt angle of the reference wave can be easily corrected during the reconstruction procedure; then the non-tilted phase distribution of the object wave *η*_Obj_ (*x*, *y*) can be obtained.

### Categorization of holography

In most electron holography, the specimen is imaged under the infocus condition on the hologram, and the reference wave is superimposed on the specimen image. Therefore, the propagation distance between the specimen and image (hologram) Δ*f*, which is often referred to as the out-of-focus distance, is effectively zero. This type of holography is called an image holography. Both ordinary micrographs and holograms can be observed and recorded in the same experiment in electron microscopy. For magnetization observation, the out-of-focus conditions for Δ*f* are adopted for Lorentz microscopy under the Fresnel mode. It is convenient to categorize optical systems for holography in terms of Δ*f*. Table 1 shows the categorization of the holography.

**Table 1:** Categorization of holography in terms of the propagation distance Δ*f* between object and hologram

**Classified name**	**Propagation distance: Δ*f***	**Features**
Image holography (conventional electron holography)	Δ*f* = 0	• Hologram positions at the same plane of the object or object image
		• Hologram is an object image superimposed of interference fringes, named interferogram or image hologram
		• Hologram has no imaging ability
Fresnel holography	Δ*f* ≤ *d*^2^/*λ*	• The distance between object and hologram is relatively short
		• The object wave is a Fresnel diffraction wave
		• Hologram has an imaging ability
Fraunhofer holography^*^	Δ*f* >> *d*^2^/*λ* (*d*: target size)	• The distance between object and hologram is sufficiently long corresponding to the Fraunhofer region
		• The object wave is a Fraunhofer diffraction wave
		• Hologram has an imaging ability
Fourier transform holography*	Δ*f* = ∞	• The distance between object and hologram is infinite
		• The object wave is a diffraction wave generated by using a Fourier transform lens
		• Reconstruction requires only one Fourier transform
Lens-less Fourier transform holography	Δ*f* = ∞	• Point source of reference wave is positioned on the same plane with the object plane
		• Even mechanical propagation distance is small, an optical Δ*f* is infinite; then Fourier transform holography is realized
		• Reconstruction requires only one Fourier transform

^*^ For the Fraunhofer holography and Fourier transform holography, the diffraction waves are localized in a narrow spatial region; therefore, it is difficult to observe and record interference fringes as holograms.

## Coherence of electron waves

An electron microscope is a scientific instrument developed under the assumption that electrons can be treated as waves; furthermore, electron holography requires that phases of electron waves can be defined and their distributions can be recorded as interference fringes. Generally, a spatially spread plane wave with a uniform phase value, such as an equi-phase plane, is called a wavefront. Widely spread wavefronts are advantageous to form interferences. In this section, we discuss the relationships between the spread of the wavefront and coherence of the waves.

### Spatial coherence

**Fig. 2:**
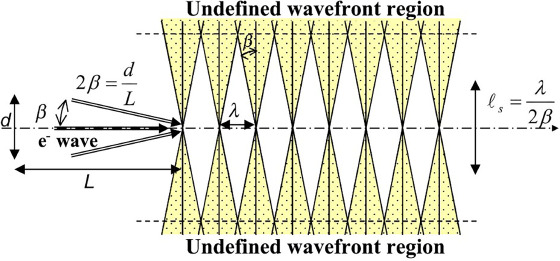
Relation between spatial coherence and divergence angle of plane waves.

Figure 2 shows a schematic diagram in which one plane wave propagating from left to right on the optical axis and two plane waves propagating with the tilt angle (semi-divergence angle) of ±*β* to the optical axis are superimposed. The equi-phase planes of three waves are aligned just on the optical axis at the distance of the wavelength *λ*. On and around the optical axis, these three waves can be drawn as one wavefront, but the equi-phase planes of tilted plane waves deviate away from the optical axis in the vertical direction. Then, at places with the distance from the optical axis larger than the distance *l*_s_, the front and back equi-phase planes of the tilted plane waves are mixed, and the wavefront can no longer be definable This distance in which the wavefront can be defined is called the spatial coherence length *l*_s_, given by [1, 2]

(5) \begin{equation*} {l}_{\mathrm{s}}=\frac{\lambda }{2\beta }. \end{equation*}

For example, for the electron wave accelerated at 300 kV, with the wavelength *λ* of 2 × 10^−3^ nm and the divergence angle 2*β* of 1 × 10^−5^ rad, the spatial coherence length *l*_s_ becomes 200 nm. The optical system, including experimental devices and their setup for electron interferometry, must be designed so that interference experiments should be performed within this relatively small spatial size *l*_s_.

### Temporal coherence

**Fig. 3:**
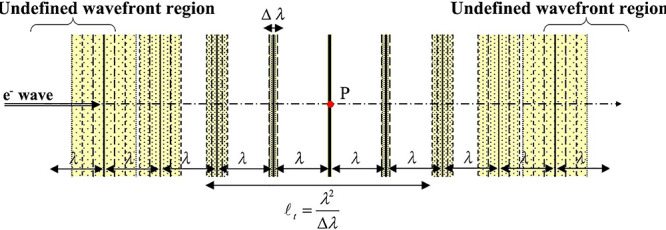
Relation between temporal coherence and deviation of wavelength of plane waves.

Figure 3 shows a schematic diagram in which one plane wave propagates along the optical axis with a spread in the wavelength Δ*λ*. When the distance from the point P at the center of the optical axis to the front or back of the propagation direction is increased, the equi-phase planes are mixed due to the difference in wavelength *λ* ± Δ*λ*. Then, a distance farther than a distance *l*_t_, the front and back equi-phase planes of the plane waves are mixed, and then the wavefront can no longer be defined. This distance in which the wavefront can be defined is called the temporal coherence length *l*_t_ given by [1, 2]

(6) \begin{equation*} {l}_{\mathrm{t}}=\frac{\lambda^2}{\Delta \lambda }\ . \end{equation*}

Since conventional electron microscopes are equipped with stable power supplies for electron beam acceleration and lens current control with deviation ratio of the order of 10^−6^, this temporal coherence length (*l*_t_ ~ 10^−6^ × *λ*) is much larger than the observation specimen size.

## Wave/particle duality

Experiments on the ‘wave/particle duality’ are well known as experiments showing the essence of quantum physics [29, 30] and have been carried out repeatedly since the electron wave interference became possible [31–35]. Recent technological innovations enable us to perform these experiments with higher precision [36–39]. The ‘gedankenexperiment’ by famous theorists, such as Feynman [29] and Tomonaga [30], has now been realized as a real experiment using the state-of-the-art electron microscopes [23, 24].

### Single-electron buildup experiment

**Fig. 4:**
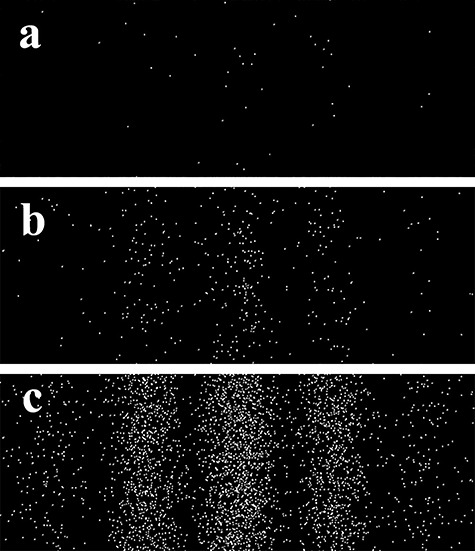
Single-electron buildup experiment: (a) 41 electrons were counted in 0.1 s in the viewing area; (b) ~400 electrons in 1.0 s and (c) ~4000 electrons in 10.0 s. The experiment was performed using the 1.2-MV field-emission transmission electron microscopy (TEM) equipped with a single-electron detector (a DED camera system).

Figure 4 shows an example of the double-slit interference fringe formation processes, i.e. a ‘single-electron buildup’ experiment [38]. Each bright spot in the figures shows an arrival point of a single electron. Figure 4a shows a single shot image for the exposure time of 0.1 s with 41 recorded electrons in this region. The number of electrons in Fig. 4b is about 400 in 1 s and that in Fig. 4c is about 4000 in 10 s. Interference fringes can easily be visualized in Fig. 4c, and even the fringes in Fig. 4a and b can be vaguely recognizable when closely watched. These results show that the highly coherent electron waves can be generated by the 1.2-MV field-emission transmission electron microscopy (TEM) [24, 40].

### Double-slit experiment depending on propagation distance

**Fig. 5:**
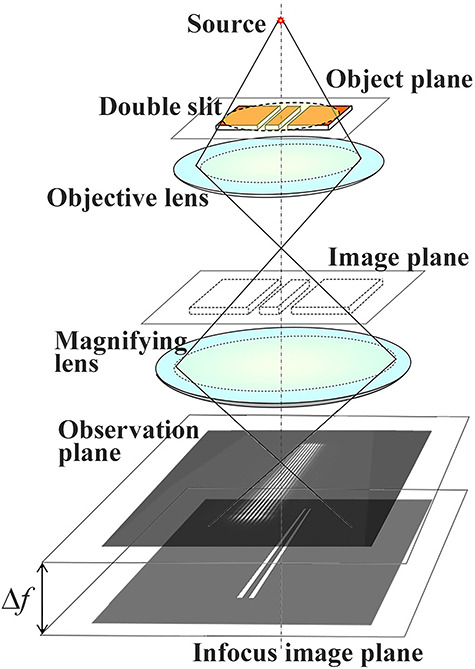
Schematic diagram of the optical system for the double-slit experiment. The double slit is placed at the specimen position, and observation of the interference fringes is controlled by using a magnifying lens with the propagation distance Δ*f*.

As indicated in Fig. 1 and Table 1, the propagation distances Δ*f* from the object to hologram strongly affect interferences of two waves. To study the dependence of the interference fringes on the propagation distance, the formation of the interference fringes was investigated by changing Δ*f* from the double-slit (specimen position) to the observation plane. Figure 5 shows the optical system setup on the 300-kV field-emission TEM, in which Δ*f* was controlled by using a magnifying lens under the objective lens [41].

**Fig. 6:**
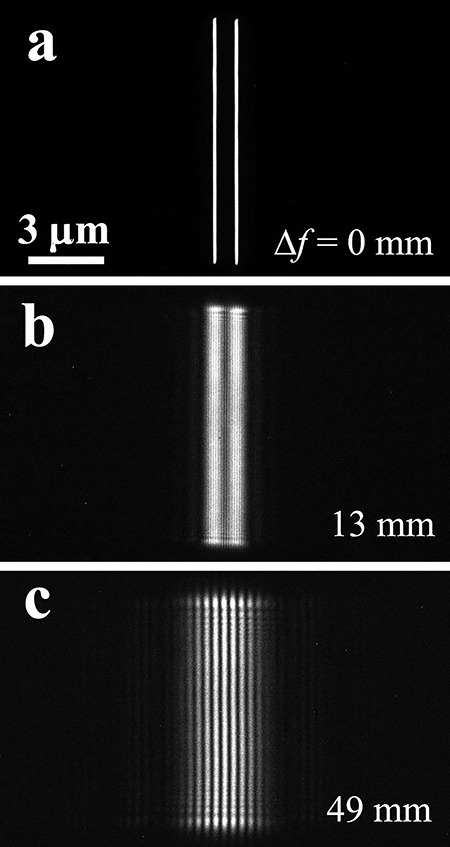
Double-slit interference experiment depending on the propagation distance Δ*f*: (a) Δ*f* = 0 mm; (b) Δ*f* = 13 mm and (c) Δ*f* = 49 mm. Opening width of the double slit is 0.12 μm in each, slit length is 10 μm and spacing between the slits is 0.8 μm.

Figure 6 shows the double-slit interference fringe images as a function of Δ*f* [41]. Figure 6a is the infocus image of the double-slit at Δ*f* = 0 mm and Δ*f* increases to 13 mm in (b) and to 49 mm in (c). When Δ*f* increases, the interference fringes show higher contrasts with wider fringe spacing. These clear interference fringe changes indicate that electrons propagate as waves.

### Electron waves and their interaction with electromagnetic fields

Electron waves can be expressed by wave functions as described in the preface of this issue. The law of optics, described in terms of ‘propagations’, ‘diffractions’ and ‘interferences’, can be practically applied to electron waves. Therefore, many kinds of electron holography, such as Fresnel holography, image holography and Fraunhofer holography, have been developed and utilized (see Table 1). However, achieving just interference and recording holograms by using electron waves is not practical and sufficient for electron holography, because it yields only replacement experiments (follow-up experiments) of light waves. In contrast, electron holography can perform much wider measurements that have not been possible with light waves.

One of the most successful experiments is the measurement of electromagnetic fields, i.e. the measurement of scalar potential *V* and vector potential ***A*** [1, 2], which is described in detail. The time-independent Schrödinger equation under electromagnetic fields can be written as [42, 43]

(7) \begin{equation*} \frac{1}{2m}{\big(-i\mathrm{\hslash}\nabla -e\boldsymbol{A}\big)}^2\varPsi -\big(E- eV\big)\varPsi =0, \end{equation*}

where $ \mathrm{\hslash} $ is Planck’s constant (*h*) divided by 2π, *Ψ* is wave function, *m* is electron mass, −e is electron charge, *E* is electron energy, ***A*** is vector potential and *V* is scalar potential. For the sake of simplicity, we consider the behavior of electrons in a sufficiently weak electromagnetic field so that electrons are not largely deflected. Then, the Schrödinger equation can be solved by using the WKB approximation. When the wave function *Ψ* is expressed by an amplitude *Ψ*_0_ and a phase *S*/$ \mathrm{\hslash} $ as

(8) \begin{equation*} \varPsi ={\varPsi}_0\exp \left[i\frac{S}{\mathrm{\hslash}}\right], \end{equation*}

then the phase of the electron wave can be derived in a non-relativistic approximation:

(9) \begin{equation*} \frac{S}{\mathrm{\hslash}}=\frac{1}{\mathrm{\hslash}}\int \big(\boldsymbol{p}-e\boldsymbol{A}\big)\cdot \mathrm{d}\mathbf{s}=\frac{1}{\mathrm{\hslash}}\int \left(\sqrt{2m\big(E- eV\big)}-e\boldsymbol{\tau} \cdot \boldsymbol{A}\right)\mathrm{ds}. \end{equation*}

Here the integral is carried out along the electron trajectory, ***p*** is the momentum vector of an electron, and ***τ*** is the unit tangent vector of the electron trajectory. When the electron moves only in electrostatic fields (***A*** = 0), the wavefront that defines the equi-phase plane is perpendicular to the electron trajectory. On the other hand, in the magnetic fields (***A*** ≠ 0), the wavefront is not perpendicular to the trajectory but to the momentum ***p***$-$*e**A***.

The phase function of the electron wave is not determined uniquely because of the gauge freedom of the vector potential ***A***. However, a phase difference between two electron trajectories ΔS/$ \mathrm{\hslash} $ can be determined uniquely. Then, the phase difference is given by

$$\begin{eqnarray*} \Delta \frac{S}{\mathrm{\hslash}}\!\!\!\! &=&\!\!\!\! \frac{1}{\mathrm{\hslash}}{\int}_{\mathrm{I}}\left(\sqrt{2m\big(E- eV\big)}-e\boldsymbol{\tau} \cdot \boldsymbol{A}\right)\cdot \mathrm{ds} \\ &&\!\!\!\! -\frac{1}{\mathrm{\hslash}}{\int}_{\mathrm{I}\mathrm{I}}\left(\sqrt{2m\big(E- eV\big)}-e\boldsymbol{\tau} \cdot \boldsymbol{A}\right)\cdot \mathrm{ds} \end{eqnarray*}$$

(10) \begin{equation*} =\frac{1}{\mathrm{\hslash}}{\oint}_c\left(\sqrt{2m\big(E- eV\big)}-e\boldsymbol{\tau} \cdot \boldsymbol{A}\right)\cdot \mathrm{ds}, \end{equation*}

where the subscripts I and II indicate two trajectories and c means the enclosed path of the trajectories.

The acceleration voltage of the ordinary TEMs is about 100 kV or higher, and the potential of the observation targets is considered to be several tens of volt or less. Therefore, further approximation, *E* >> *eV*, is applied to Eq. (10) yielding

(11) \begin{equation*} \Delta \frac{S}{\mathrm{\hslash}}\approx{\oint}_c\left(\boldsymbol{k}-\frac{eV}{2E}\boldsymbol{k}-\frac{e}{\mathrm{\hslash}}\boldsymbol{A}\right)\cdot \mathrm{d}\mathbf{s}, \end{equation*}

where ***k*** is the wave vector with $|{\boldsymbol k}|=(\sqrt{(2\ {\text m}E)})/ \mathrm{\hslash} $. The first term gives a geometrical optical path difference exemplified in miller microscopy [44] and reflection electron holography [45, 46]; the second term indicates the phase difference given by the electrostatic field exemplified in p-n junctions [47, 48], inner potential measurements [49] and high-resolution observations [50–53], and the third term indicates the phase difference created by the magnetic field exemplified in magnetization measurements [54–56].

In particular, the third term can be rewritten from the closed loop path integral to the area integral using Stokes’ theorem

(12) \begin{equation*} \Delta{\frac{S}{\mathrm{\hslash}}}_{\mathrm{Mag}}=\frac{e}{\mathrm{\hslash}}{\oint}_c\boldsymbol{A}\cdot \mathrm{d}\mathbf{s}=\frac{e}{\mathrm{\hslash}}{\int}_{S_c}{B}_c\cdot \mathrm{d}\mathrm{S}, \end{equation*}

where *B*_c_ is magnetic flux in the closed path (loop) and *S*_c_ is the area of the closed loop. Equation (12) is related to the Aharonov-Bohm effect [16], which states that magnetic fields not directly applied to the electron waves affect the phase difference of the electron wave function. This effect was experimentally verified by many scientists [17, 57–60].

## Electron interference optical systems

### Electron biprism

The electron biprism was invented in 1955 by Möllenstedt and Düker [25], and it is an indispensable electron optical device for interference optics as a beam splitter for electron beams [61, 62]. It has the effect of separating the incident electron beam into two electron beams and deflecting them linearly in the converging direction or in the diverging direction.

**Fig. 7:**
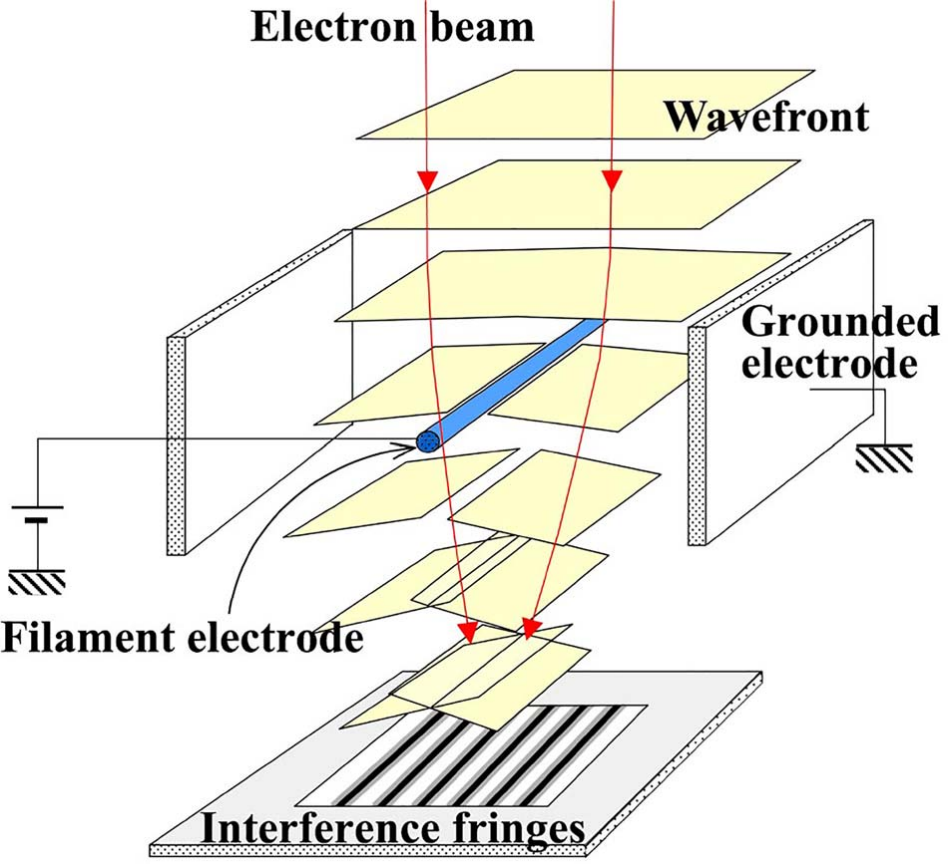
Electrostatic-type electron biprism and electron wave deflection.

As shown in Fig. 7, the electrostatic-type electron biprism is composed of a conductive filament electrode and grounded parallel-plate electrodes on both sides of the filament electrode. For the filament electrode, a metal-coated glass wire is often used with the diameter about 1 μm or less because of the small spatial coherence length of the electron waves. Figure 7 also shows how the wavefront of electron beams is deflected by the electron biprism: when a positive potential is applied to the filament electrode, the electron waves passing through on both sides of the filament electrode are deflected by the same angle in the linearly converging directions. When a negative potential is applied, the electron waves are deflected in the linearly diverging directions. The deflection angle of electron beams *α* is proportional to the potential applied to the filament electrode and independent of the incident position to the electron biprism given by

(13) \begin{equation*} \alpha ={k}_{\mathrm{f}}{V}_{\mathrm{f}}, \end{equation*}

where *V*_f_ is the applied voltage and *k*_f_ is the deflection coefficient (~10^−6^ rad V^−1^) determined by the diameter of filament electrode and the distance between the parallel plate electrodes [63]. The linear relation between *α* and *V*_f_ indicates that the electrostatic-type biprism can control electron beam deflections; therefore, this type of biprism is widely used as a practical beam splitter for electron waves.

### Single biprism interference system

**Fig. 8:**
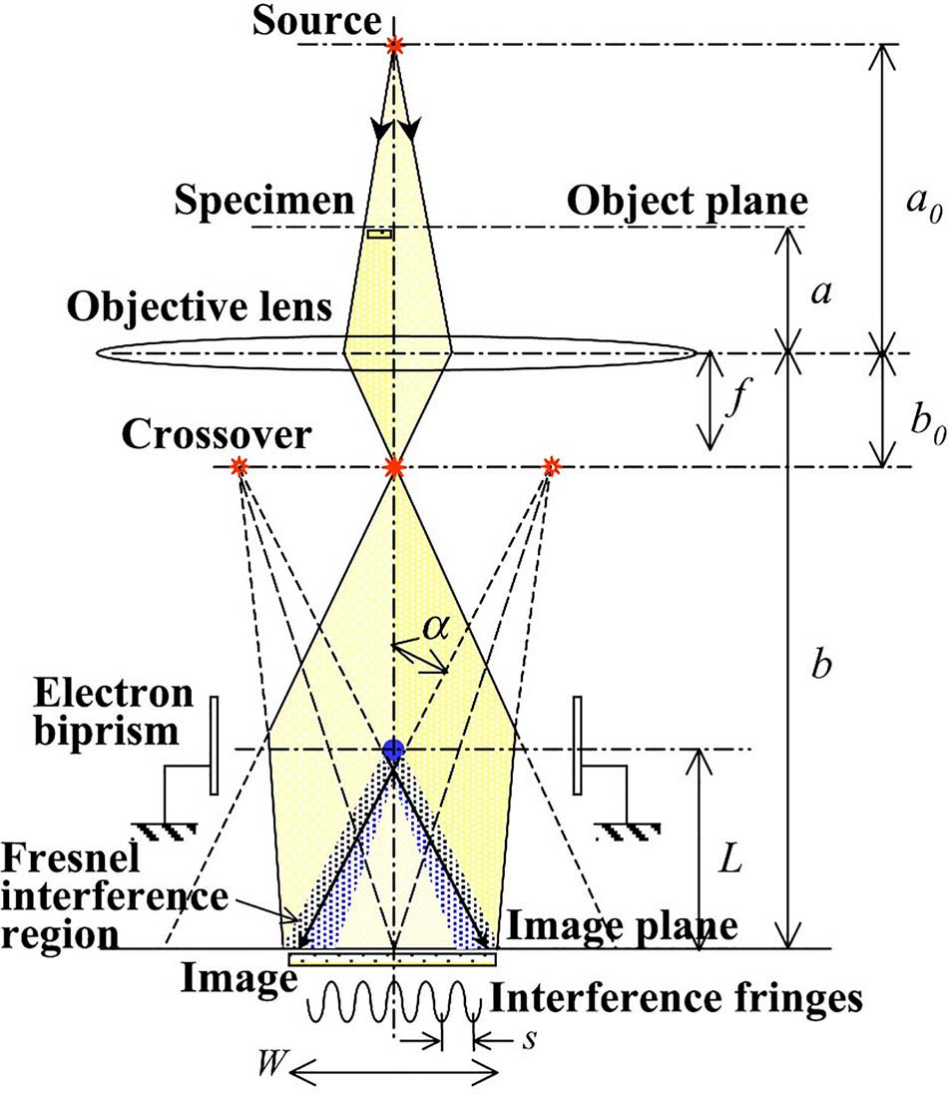
Optical system for conventional electron holography. An electron biprism is placed between the objective lens and the image plane.

Figure 8 shows a conventional optical system for hologram recording in an off-axis electron holography by using only one electron biprism placed between an objective lens and an image plane [1, 2]. When the object wave transmitted through the specimen passes through the right side of the filament electrode and the reference wave passed through the vacuum area passes through the left side of the filament electrode, an interferogram (or an image hologram) of the specimen is created on the image plane. In this interferogram the phase distribution of the object wave is recorded in the form of interference fringes with shifts. The reconstruction of the phase distribution from the image holograms requires quantitative analyses of distortions in interference fringes. For interferometry and holography analyses, fringe spacing *s* and interference width *W* are important parameters, because *s* determines the spatial resolution of the reconstructed amplitude and phase images and *W* determines the size of the observed region. Also, the contrast of the interference fringes influences the reconstructed image precision.

In the following argument, the parameters *s* and *W* are converted to *s*_obj_ and *W*_obj_ to indicate values at the specimen position (i.e. object plane position). Parameters *s*_obj_ and *W*_obj_ can be expressed in terms of as *D* and *L* as

(14) \begin{equation*} {s}_{\mathrm{obj}}=\frac{1}{M_{\mathrm{obj}}}\cdot \frac{D\lambda}{2\alpha \big(D-L\big)}, \end{equation*}

(15) \begin{equation*} {W}_{\mathrm{obj}}=\frac{1}{M_{\mathrm{obj}}}\cdot 2\alpha L-\frac{1}{M_{\mathrm{obj}}}\cdot \frac{D}{D-L}{d}_{\mathrm{f}}, \end{equation*}

where *M*_obj_ (= *b*/*a*) is the magnification of the objective lens, *α* is the deflection angle and *d*_f_ is the diameter of the filament electrode of the biprism shown by a small blue-filled circle in Fig. 8.

In this optical system, both *s*_obj_ and *W*_obj_ can be controlled easily and simultaneously by the voltage *V*_f_ applied to the filament electrode. However, for getting appropriate values of both *s*_obj_ and *W*_obj_ simultaneously, the entire optical system must be reconstructed to make these parameters suitable for different observations. In addition, this system has another disadvantage that that Fresnel diffraction waves generated by the filament electrode are superimposed on the holograms as Fresnel fringes, creating artifacts on the reconstructed amplitude and phase images. These disadvantages were solved by introducing the double biprism interferometry described in the next section.

### Double biprism interference system

**Fig. 9:**
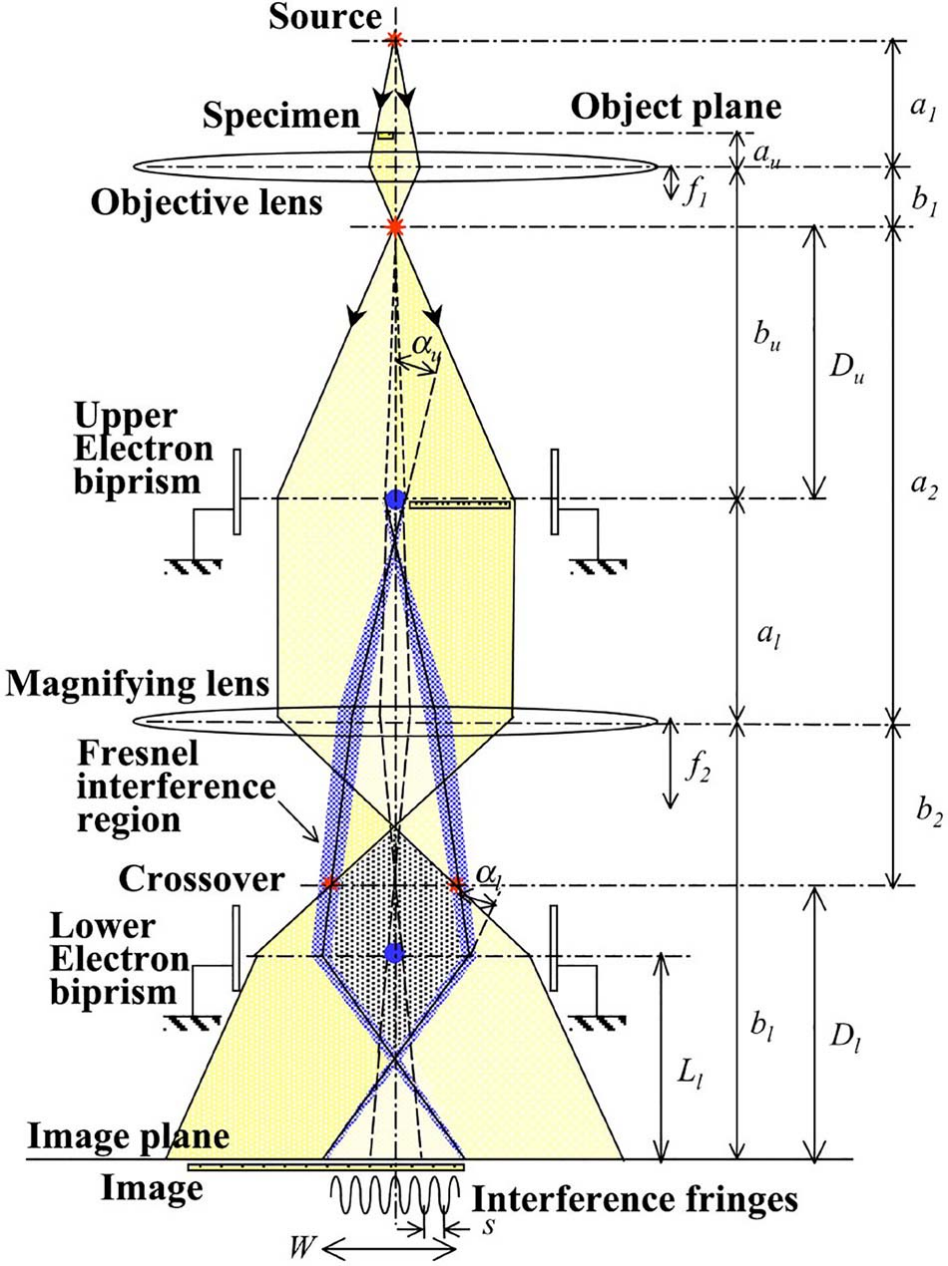
Optical system for the double biprism electron holography. An upper electron biprism is placed on the image plane of the objective lens, and a lower biprism is placed between the crossover plane of magnifying lens and image plane.

Figure 9 shows an optical system for the double biprism electron holography [64, 65]. The filament electrode of the upper electron biprism is placed just on the image plane (a real space) of the objective lens; the lower filament electrode is placed between the crossover plane of the magnifying lens (a Fourier-transformed reciprocal space) and the image plane, which is in the shadow area of the upper filament electrode. Being placed in the real space, the upper biprism can control the propagation angles of two electron waves, and being placed in the reciprocal space, the lower biprism can control the overlapping region of two electron waves. In this way it becomes possible to independently control the interference fringe spacing *s*_obj_ and the interference width *W*_obj_. The upper biprism located in the real space can control the spatial frequency, such as the interference fringe spacing, which is related to the reciprocal space, the lower biprism located in the reciprocal space can control the image position with the interference width which is related to the real space. These reciprocal controls can be considered as Fourier transform of each other because the wave propagation corresponds to Fourier transform of an object.

By using the geometrical parameters in Fig. 9, the interference parameters *s*_obj_ and *W*_obj_ can be expressed as

(16) \begin{equation*} {s}_{\mathrm{Obj}}=\frac{1}{M_l}\frac{1}{M_u}\frac{\alpha_2{D}_l\lambda }{2\big[{\alpha}_l{a}_2\big({D}_l-{L}_l\big)+{\alpha}_u{b}_2{D}_u\big]}, \end{equation*}

(17) \begin{equation*} {W}_{\mathrm{Obj}}=\frac{1}{M_l}\frac{1}{M_u}2{\alpha}_l{L}_l-\frac{1}{M_u}{d}_{\mathrm{ufi}}, \end{equation*}

where *d*_ufi_ is the diameter of the upper filament electrode. To control *s*_obj_ and *W*_obj_ independently, the following operating procedure must be taken:

(i) Determine the interference width *W*_obj_ by using the lower biprism (*s*_obj_ is optional).

(ii) Correct *s*_obj_ to an appropriate value by using the upper biprism (*W*_obj_ does not change).

Independent control of these two parameters *s*_obj_ and *W*_obj_ is effective in obtaining reconstructed amplitude and phase images for high-resolution and high-precision observation.

Since the upper filament electrode is placed just on the image plane of the object lens, the Fresnel diffraction waves generated by the filament are imaged only on the edges of the filament image, i.e. on the edges of the interference region, and thus the Fresnel diffraction fringes are not superimposed on the holograms. Although several methods have been developed to compensate the influence of Fresnel fringes on holograms in ordinary electron holography, the double biprism holography in principle does not create Fresnel fringes on the holograms. Therefore, the present method can be used for highly accurate phase distribution measurements. Furthermore, this method can be applied to the phase shift reconstruction.

**Fig. 10:**
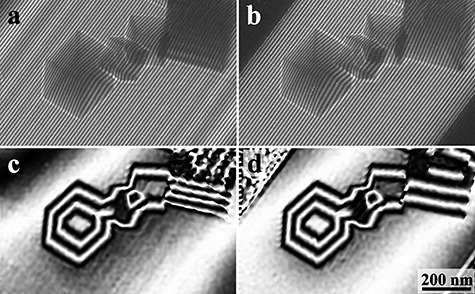
Interferograms (holograms) and reconstructed phase images of MgO fine particles: (a) hologram by the conventional system; (b) hologram by the double biprism system; (c) reconstructed phase image from (a) and (d) reconstructed phase image from (b).

Figure 10 shows interferograms (holograms) of small crystalline particles of MgO as an example of observation results using the double biprism interferometer. Figure 10a is a hologram by using the conventional holography system, and Fig. 10b is a hologram by using the double biprism system. Figure 10c is a reconstructed phase image from (a), and Fig. 10d is a reconstructed phase image from (b). Since the phase modulation due to the Fresnel fringes seen in Fig. 10c does not appear in (d) at all, the double biprism holography is effective for improving the accuracy of the conventional holography.

### Split illumination interference system

Electron holography can be realized only within the spatial coherence length of electron waves. In the present holography optical system, the coherence length can be taken to be about 10 times the diameter of the filament electrode on the first image plane for the specimen. Then, the object wave and the reference wave can be set close to each other within this coherence length. This restriction is partially but effectively eliminated by the split illumination holography [66, 67]. Although this method does not improve the degree of electron wave coherence, it has been attracting attention as a method for practically solving problems due to lack of coherence length of electron wave.

**Fig. 11:**
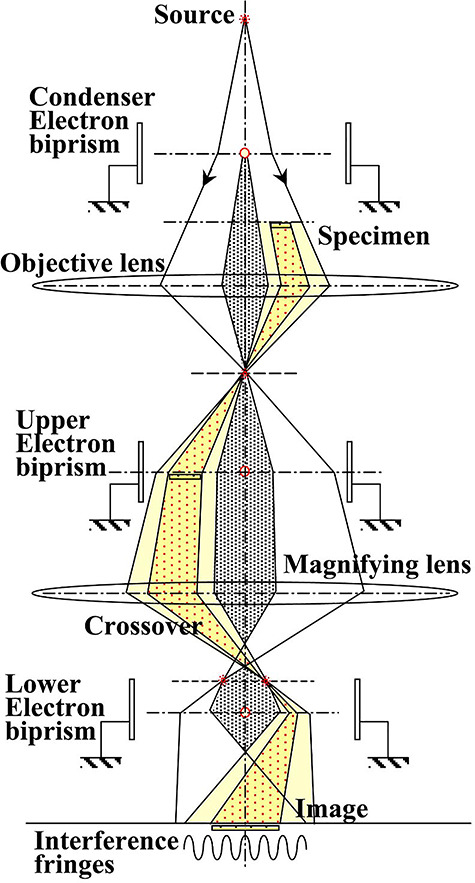
Optical system for the split illumination electron holography.

Figure 11 shows an optical system of the split illumination electron holography. Since this system uses the double biprism optical system discussed in the previous section, all three electron biprisms are shown. The condenser biprism placed above the specimen separates the irradiating electron wave into two waves; the object wave drawn on the right-hand side irradiates the distant regions on the object plane, and the reference wave is drawn on the left-hand side. In this way, holography observation becomes possible even in the region far away from the specimen edge. In addition, the reference wave is allowed to pass the region far away from the specimen edge, where leaked magnetic fields from the specimen are sufficiently small. With this system highly accurate observations of electromagnetic fields will become possible. The optical system may cause Fresnel fringes due to the condenser biprism to be superimposed on the holograms, but this can be eliminated by using a double biprism optical system for irradiation system [67].

The double biprism holography and the split illumination holography are expected to be the mainstream of the interference microscopy in the near future, and the double biprism holography microscopes are getting popular in the world.

### Other optical systems of electron holography

We have so far discussed the electron holography under infocus conditions by using the electron biprisms in the wavefront-division type; however, several other systems of electron holography have also been developed. In this section, we briefly discuss some of them.

#### Amplitude-division type holography

An amplitude-division-type electron interferometry, a Mach-Zehnder-type electron interferometry [68–74], was developed before the invention of the electron biprism [68, 69]. Two or three single-crystalline thin films are used as beam splitters instead of half-mirrors in the light optics. This type of interferometers requires less restriction on the spatial coherence length than the wavefront-division-type interferometer does, and therefore, thermal emission electron beams can be used [72]. Since single-crystalline thin films had to be placed in narrow gaps having a few micrometers, it was difficult to place specimens or optical elements in these narrow gaps, making it difficult for practical use. Very recently, an electron optical system including an imaging lens between thin films has been developed, but its practical realization will take some time [74].

#### Scanning-type holography

An electron beam is split into two or more beams in the condenser optical system above the specimen, and beam spots are formed on the specimen position. Two selected beam spots are scanned on and around the specimen simultaneously. Then, those beams are superimposed and interfered with each other by the imaging optical system placed below the specimen position. The phase distribution of the object wave passed through the specimen can be directly obtained by drawing the fringe position displacements of the interference fringes in correlation with the scanning position of the specimen [75, 76].

#### Fraunhofer holography and lens-less Fourier transform holography

These methods are effective for a large propagation distance between the spatial position for the target object and the position for recording hologram [77–80]. It is particularly effective for the electron holography when small-angle electron diffractions [81] are involved, for example, observations of vortex beams [82], magnetic materials and diffraction waves.

**Fig. 12:**
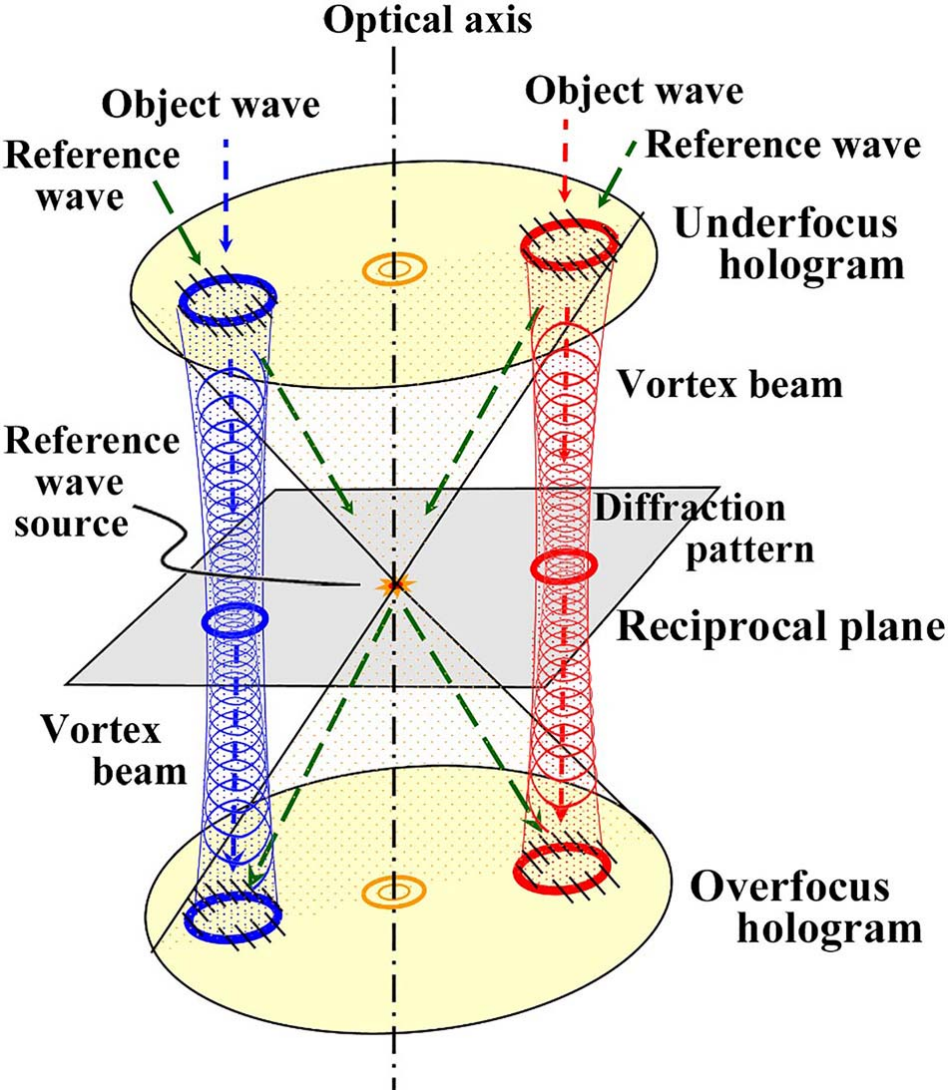
Optical system of lens-less Fourier transform holography. Two vortex beams (blue and red) from a fork-shaped grating as object waves at the reciprocal plane and a spherical transmitted wave as the reference wave (green broken arrows) generate a source spot just on the optical axis.

**Fig. 13:**
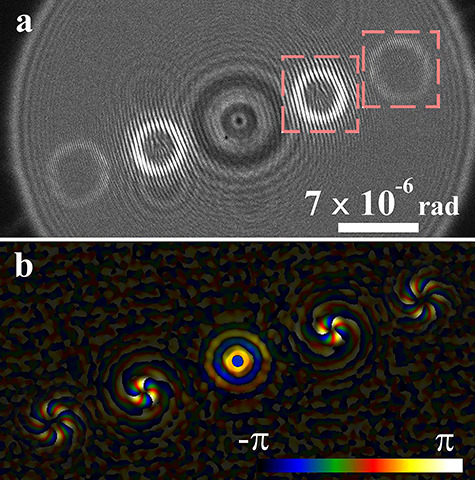
(a) Lens-less Fourier transform holograms of four vortex beams, and (b) composite image of reconstructed amplitude and phase distributions from the first- and second-order diffraction waves shown in the squares with red broken lines on the right-hand side of (a).

As an example, we discuss phase distribution observation of vortex beams generated by fork-shaped gratings [83–86] by using lens-less Fourier transform holography [79, 80]. Figure 12 shows an experimental setup [80]: Bragg diffraction waves as the vortex beams from the grating correspond to the object waves, and a transmitted spherical wave along the optical axis corresponds to a reference wave. A reference wave source positioned on the same plane as that of specimens is a typical structure of lens-less Fourier transform holography. Two-wave interference patterns are recorded away from the reciprocal plane without the electron biprism. Since interferograms generated by the object and reference waves are recorded in angular patterns after a long propagation, the amplitude and phase images were reconstructed through only one Fourier transformation procedure. Figure 13a shows the underfocus hologram of diffraction spots of the vortex beams, and Fig. 13b shows composite images of amplitude and phase images for the first- and second-order diffraction spots of the right-hand side of (a). The phase distributions are shown in the color-coded mode combined with amplitude distributions with the luminance [80].

## Reconstruction systems

The reconstruction system of electron holography is closely related to the recording optical systems for holograms. For example, in the phase shift method described below, it is necessary to change the phase of the reference wave or the object wave while keeping the other optical conditions, such as the specimen position and its focus constant. To satisfy these conditions with high accuracy, special attention must be paid in constructing the hologram recording system.

### Optical method

An optical reconstruction method is the direct implementation of Gabor’s original holography idea in which the wavefronts of electron waves are replaced by those of light waves [1, 2]. The electron holograms are regarded as diffraction gratings, and laser lights are irradiated on them as reconstruction waves. The plus-first-order or minus-first-order diffraction waves passed through the electron hologram are separated spatially and selected by using some optical devices. Then, the electron object wave is reconstructed as a light wave to obtain the virtual or real object images. As described above, this method is so cumbersome that it is no longer used and now is replaced by the Fourier transform method, thanks to the rapid progress in computer capabilities and improvement of image processing techniques.

### Fourier transform method

In the Fourier transform method, the optical reconstruction procedures discussed in Section 6.1 are performed by computer algorithm [87–90]. This method is the most common method for reconstructing the electron holograms now.

**Fig. 14:**
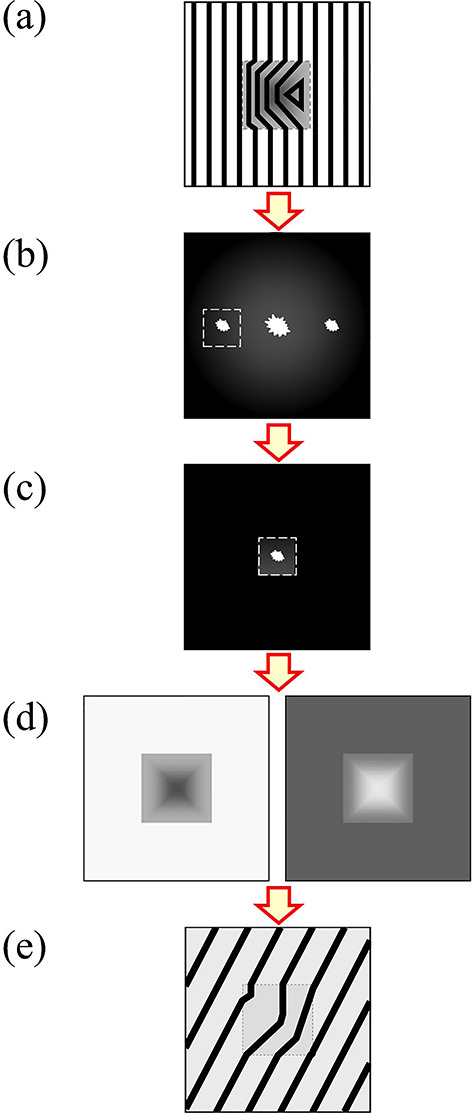
Procedure of Fourier transform reconstruction method: (a) interferogram (hologram); (b) power spectrum; (c) sideband after trimming; (d) reconstructed amplitude image and phase image and (e) interferogram.

Figure 14 shows the procedure of Fourier reconstruction method. Figure 14a depicts an input hologram (interferogram), where interference fringes are superimposed on a rectangular particle having a pyramid shape due to fringe shifts. Figure 14b shows a Fourier transform image of (a). A star-shaped white distribution at the center is the power spectrum of the particle image, and two spots on the left side and right side are sideband spots corresponding to the diffraction spots from the hologram of (a). One of the sideband spots corresponds to the object wave for a real image, and other sideband spot corresponds to the conjugate wave for a virtual image. The numerical data of both sideband spots contain the amplitude and phase information. One of the sideband spots in (b) is selected and moved to the center of the calculation region. Figure 14c shows the left sideband spots moved to the center. Using the inverse Fourier transformation on (c), an amplitude distribution [left-hand side in (d)] and a phase distribution [right-hand side in (d)] are obtained from the real part and imaginary part of the numerical data, respectively. Phase distribution images (interferograms) are often displayed in sinusoidal functions. Figure 14e shows the interferogram in equi-phase line images with different directions.

Mathematical procedures of the Fourier transform method are as follows: intensity distribution of electron hologram *I* (*x*, *y*), Eqs. (3) and (4), in the exponential form is given by

(18) \begin{equation*} I\big(x,y\big)={\big|{\varphi}_0\big|}^2+1+{\varphi}_0{e}^{i\big({\eta}_0\big(x,y\big)-2\pi{R}_0x\big)}+{\varphi}_0{e}^{-i\big({\eta}_0\big(x,y\big)-2\pi{R}_0x\big)}. \end{equation*}

This corresponds to input image data shown in Fig. 14a. Fourier transform of Eq. (18), *F* [*I*], is given by

(19) \begin{equation*} F\big[I\big]\big({R}_x,{R}_y\big)=\boldsymbol{A}\big({R}_x,{R}_y\big)+\boldsymbol{B}\big({R}_x-{R}_0,{R}_y\big)+{\boldsymbol{B}}^{\ast}\big({R}_x+{R}_0,{R}_y\big), \end{equation*}

(20) \begin{equation*} \left\{\begin{array}{c}\boldsymbol{A}\big({R}_x,{R}_y\big)=F\big[{\big|{\varphi}_0\big|}^2+1\big]\big({R}_x,{R}_y\big)\\[6pt] {}\boldsymbol{B}\big({R}_x-{R}_0,{R}_y\big)=F\big[{\varphi}_0{e}^{i{\eta}_0\big(x,y\big)}\big]\big({R}_x-{R}_0,{R}_y\big)\\[6pt] {}{\boldsymbol{B}}^{\ast}\big({R}_x+{R}_0,{R}_y\big)=F\big[{\varphi}_0{e}^{-i{\eta}_0\big(x,y\big)}\big]\big({R}_x+{R}_0,{R}_y\big)\end{array}\right\}, \end{equation*}

where (*R*_x_, *R*_y_) are the coordinate axes in the reciprocal space and *R*_0_ is the spatial carrier frequency, which is the reciprocal of the averaged interference fringe spacing. Equation (19) corresponds to Fig. 14b. When the three terms of Eq. (19), i.e. Eq. (20), can be separated by the carrier spatial frequency *R*_0_, each term can be calculated independently. For the separation of these terms, the spreads of the distributions of terms ***A*** must be two-third or less than *R*_0_, and those of ***B*** and B^*^ must be one-third or less than *R*_0_. Therefore, three times the interference fringe spacing {1/(3*R*_0_)} becomes the maximum resolution of the reconstructed amplitude and phase images in this method.

If the term ***B*** is selected and the coordinates are moved by *R*_0_ to the origin of the calculation region, we obtain:

(21) \begin{equation*} \boldsymbol{B}\big({R}_x,{R}_y\big)=F\big[{\varphi}_0{e}^{i{\eta}_0\big(x,y\big)}\big]\big({R}_x,{R}_y\big), \end{equation*}

which corresponds to the data shown in Fig. 14c.

When Eq. (21) is inverse Fourier transformed, we obtain:

(22) \begin{equation*} \hat{I}\big(x,y\big)={\varphi}_0\big(x,y\big){e}^{i{\eta}_0\big(x,y\big)}={\Phi}_0\big(x,y\big), \end{equation*}

(23) \begin{equation*} =\hat{I_R}\big(x,y\big)+i\hat{I_I}\big(x,y\big). \end{equation*}

Here *Î*_R_ (*x*, *y*) and *Î*_I_ (*x*, *y*) are the real part and imaginary part of $\hat{I}\big(x,y\big)$.Then the amplitude distribution ${\varphi}_0(x,y)$and the phase distribution ${\varphi}_0(x,y)$ are obtained as

(24) \begin{equation*} {\varphi}_0\big(x,y\big)=\sqrt{\hat{I_R}{\big(x,y\big)}^2+\hat{I_I}{\big(x,y\big)}^2}, \end{equation*}

(25) \begin{equation*} {\eta}_0\big(x,y\big)={\tan}^{-1}\frac{\hat{I_I}\big(x,y\big)}{\hat{I_R}\big(x,y\big)}. \end{equation*}

When the numerical data based on Eqs. (24) and (25) are obtained, interferograms are created through numerical processing. In general, the interferograms are displayed in the cosine form for phase distribution excluding the value of amplitude distribution as

(26) \begin{equation*} \cos{\eta}_0\big(x,y\big)=\frac{\hat{I_R}\big(x,y\big)}{\sqrt{\hat{I_R}{\big(x,y\big)}^2+\hat{I_I}{\big(x,y\big)}^2}}. \end{equation*}

This corresponds to the interferogram shown in Fig. 14e.

### Phase shift method

Electron holograms are composed of specimen images superimposed with the interference fringes, such as an interferogram, so that several fringe analysis methods have been developed to obtain holograms [91–93]. In particular, the phase shift reconstruction method is now most commonly used for achieving high-resolution and high-precision electron hologram data. Its features are as follows: (i) it does not depend on the interference fringes and their displacements in one hologram and (ii) it uses a number of holograms with a known phase difference between the object and reference waves [94–98].

The *m*th hologram within the total number of *M* holograms *I*_H_ (*x*, *y*; *m*) can be expressed by

(27) \begin{equation*} {I}_{\mathrm{H}}\big(x,y;m\big)=a\big(x,y\big)+c\big(x,y\big){e}^{i\varphi (m)}+{c}^{\ast}\big(x,y\big){e}^{- i\varphi (m)}, \end{equation*}

(28) \begin{equation*} \left\{\begin{array}{c}a\big(x,y\big)={\big|{\varphi}_0\big(x,y\big)\big|}^2+1={I}_0\big(x,y\big)+1\\[6pt] {}c\big(x,y\big)={\varphi}_o\big(x,y\big)\mathit{\exp}\big[i\big({\eta}_o\big(x,y\big)\hbox{-} 2{\pi R}_{0x}x\hbox{-} 2{\pi R}_{0y}y\big)\big]\\[6pt] {}{c}^{\ast}\big(x,y\big)={\varphi}_o\big(x,y\big)\mathit{\exp}\big[\hbox{-} i\big({\eta}_o\big(x,y\big)\hbox{-} 2{\pi R}_{0x}x\hbox{-} 2{\pi R}_{0y}y\big)\big]\end{array}\right\} \end{equation*}

Equation for three unknown functions, *a* (*x*, *y*), *c* (*x*, *y*) and c^*^(*x*, *y*), can be expressed in the matrix form as

(29) \begin{equation*} \big(1\kern0.5em \exp \big[ i\varphi (m)\big]\kern0.5em \exp \big[- i\varphi (m)\big]\big)\left(\begin{array}{c}a\big(x,y\big)\\{}c\big(x,y\big)\\{}{c}^{\ast}\big(x,y\big)\end{array}\right)={I}_H\big(x,y;m\big). \end{equation*}

Then, *a* (*x*, *y*), *c* (*x*, *y*) and c^*^(*x*, *y*) can be obtained by using the inverse matrix after expanding Eq. (29) and summing over m:

$$ \left(\begin{array}{@{}c@{}}a\big(x,y\big)\\{}c\big(x,y\big)\\{}{c}^{\ast}\big(x,y\big)\end{array}\right)= $$

(30) \begin{eqnarray*} &&{\left(\!\!\begin{array}{@{}c@{\quad\!\!}c@{\quad\!\!}c@{}}M& \sum_{m=1}^M\exp \big[ i\varphi (m)\big]& \sum_{m=1}^M\exp \big[- i\varphi (m)\big]\\[6pt] {}\sum_{m=1}^M\exp \big[- i\varphi (m)\big]& M& \sum_{m=1}^M\exp \big[-2 i\varphi (m)\big]\\[6pt] {}\sum_{m=1}^M\exp \big[ i\varphi (m)\big]& \sum_{m=1}^M\exp \big[2 i\varphi (m)\big]& M\end{array}\!\!\right)}^{-1} \nonumber \\[3pt] && \times \left(\begin{array}{@{}c@{}}\sum_{m=1}^M{I}_H\big(x,y;m\big)\\[6pt] {}\sum_{m=1}^M{I}_H\big(x,y;m\big)\exp \big[- i\varphi (m)\big]\\[6pt] {}\sum_{m=1}^M{I}_H\big(x,y;m\big)\exp \big[ i\varphi (m)\big]\end{array}\right) \end{eqnarray*}

If the relative phase difference in the series holograms is regularly divided into an equal phase deference and within 2*π*, the summation over m in the off-diagonal terms in the first matrix of the right-hand side of Eq. (30) becomes zero, reducing this matrix proportional to unit matrix. In other cases, inverse matrices must be calculated. Then, the distributions of intensity *I*_0_ (*x, y*), amplitude *φ*_o_ (*x*, *y*) and phase *η*_o_ (*x*, *y*) can be expressed as

(31) \begin{equation*} {I}_0\big(x,y\big)=a\big(x,y\big)-1, \end{equation*}

(32) \begin{equation*} {\varphi}_0\big(x,y\big)=2\sqrt{c\big(x,y\big)\times{c}^{\ast}\big(x,y\big)}, \end{equation*}

(33) \begin{eqnarray*} {\eta}_0\big(x,y\big)-2\pi{R}_{0x}x-2\pi{R}_{0y}y-2 m\pi\!\!\!\!\!\! &=&\!\!\!\!\!\! {\tan}^{-1}\left[\frac{\operatorname{Im}\big[c\big(x,y\big)\big]}{\operatorname{Re}\big[c\big(x,y\big)\big]}\right] \nonumber \\ \!\!\!\!\!\!&=&\!\!\!\!\!\! {\tan}^{-1}\left[\frac{\operatorname{Im}\big[{c}^{\ast}\big(x,y\big)\big]}{\operatorname{Re}\big[{c}^{\ast}\big(x,y\big)\big]}\right].\nonumber \\ \end{eqnarray*}

Observation techniques, such as a single-particle analysis method that can analyze large numbers of images, have already been practically demonstrated their ability to reduce noises in reconstructed images and improve resolutions and accuracy in the analyses. In electron holography, these techniques using very large number of holograms have already been implemented [99]. The phase shift method is expected to be further advanced as a reconstruction method from a large number of holograms and as an interferometry in conjunction with a new hologram recording procedure.

## Conclusion

In this paper, we have reviewed the technical items for the implementation of electron holography, such as physics of electron waves, interference optics and interferometry, and reconstruction methods; treatments of phase distributions are discussed in detail. Properties and features of electron waves can be understood by using analogy with those of light waves, and electron holography can be considered as phase-division-type interferometry using electron biprism. Electron holography, however, is not only used for verification of experiments done by light-wave interferometry, but for new experiments that can be realized only by electron waves, such as observation of electromagnetic fields, magnetic domain walls and superconducting magnetic flux quanta, and confirmation experiments of the Aharonov-Bohm effect. Furthermore, electron waves applied to analyze quantum physical features (experiments), for example, the double-slit experiments, were discussed.

We hope that the readers of this paper will get a lot of information on electron holography techniques and that this paper plays an appropriate introduction to other papers in this special issue of Microscopy.
